# Understanding herpes zoster vaccine hesitancy and information asymmetry: a qualitative study in China

**DOI:** 10.3389/fpubh.2024.1429522

**Published:** 2024-09-02

**Authors:** Xiaolong Wang, Yufei Xing, Enming Zhang, Zhengyue Dai, Yuan Li, Shuhui Shang, Jiale Hu, Xian Zhang, Qiong Fang

**Affiliations:** ^1^School of Nursing, Shanghai Jiao Tong University, Shanghai, China; ^2^Department of Nurse Anesthesia, College of Health Professions, Virginia Commonwealth University, Richmond, CA, United States; ^3^Department of Nursing, Caohejing Community Health Service Center, Shanghai, China

**Keywords:** vaccine hesitancy, information asymmetry, herpes zoster, 5C, qualitative

## Abstract

**Background:**

Herpes zoster is more prevalent among the older adult due to the age-related immune decline, leading to significant pain and complications. Although vaccination effectively prevents viral infections, vaccine hesitancy remains a major barrier to achieving high vaccination rates.To address this, we conducted a qualitative survey using Vaccine Hesitancy Determinants Matrix and 5C model to understand and improve vaccination rates in this group.

**Methods:**

Descriptive qualitative research design based on the philosophical underpinnings of naturalistic inquiry and purposive sampling methodology was conducted on adults aged 50 and above, as well as community health workers. Data were collected through semi-structured, in-depth personal interviews. The interview outline was constructed following a comprehensive review of the literature and consideration of the theoretical framework.

**Results:**

Seventeen adults over 50 years and four community healthcare workers were included in this study. The study found that information asymmetry in immunization planning was evident at all stages of vaccine supply, dissemination and demand. The main manifestations included limited access to authoritative information, insufficient community awareness of herpes zoster as a route of vaccination, insufficient vocational training, significant gaps in vaccine knowledge, and high levels of complacency among individual residents.

**Conclusion:**

Herpes zoster vaccine hesitancy is prevalent among middle-aged and older adults in China due to information asymmetry, vaccine complacency, inadequate community services, and other multiple layers of factors. Public health strategies should aim to reduce cognitive biases and information gaps by disseminating diverse and credible vaccine information through social media, medical institutions, and offline channels to promote higher vaccination rates.

## 1 Introduction

In recent years, the incidence of herpes zoster, particularly among the older adult, has been on the rise. Advanced age is a major risk factor for the reactivation of the varicella-zoster virus (1). Globally, the incidence ranges from 3 to 5 per 1,000 person-years in the general population, increasing to 5 to 11 per 1,000 person-years in those aged 50 years and older (2). Apart from acute-phase lesions and pain, 9 to 34% of patients face the potential risk of developing postherpetic neuralgia (3). Overall, the healthcare burden of herpes zoster is substantial. A study by Kawai et al. found that the annual rate of herpes zoster-related hospitalizations ranges from 2.1 to 25.0 per 100,000 individuals, with hospitalization rates increasing with age (2). Additionally, the economic burden following infection is significant (4), with the average hospitalization cost for adults aged 50 and above estimated at 4,502.4 RMB (approximately $630) (5).

Vaccination against herpes zoster is an effective and feasible means of preventing infection with the herpes zoster virus and its serious complications (6). The U.S. FDA approved the recombinant herpes zoster vaccine in 2017 for preventing herpes zoster in adults aged 50 and above. It was officially launched in China in June 2020, becoming the only approved herpes zoster vaccine in this country (7). However, herpes zoster vaccination rates in China, even in cities like Shanghai (8) and Beijing (9), are strikingly low. Recent data from the past 2 years indicate that the vaccination rate for HZV among adults aged 50 years and older is less than 3.0%, significantly lagging behind some developed countries like the United States (25.8%) (10) and Greece (20.0%) (11). In China, a dedicated herpes zoster (HZ) vaccination program specifically for older adult individuals or those with weakened immune systems has not yet been established, and the HZ vaccine is not part of the mandatory immunization schedule. Despite the increasing recognition of the importance of vaccination in preventing HZ and its complications, the implementation of widespread vaccination programs remains limited.

Understanding factors associated with herpes zoster vaccine hesitancy among middle-aged and older adult individuals is crucial for improving vaccination rates in these key populations. The Measuring Behavioral and Social Drivers of Vaccination (BeSD) working group defines vaccine hesitancy as *delay in acceptance or refusal of vaccination despite availability of vaccination services. Vaccine hesitancy is complex and context specific,* var*ying across time, place and vaccines* (12, 13). Vaccine hesitancy is notably context-specific and complex, given the diversity of vaccine types and settings. The Strategic Advisory Group of Experts introduced the Vaccine Hesitancy Determinants Matrix, which thoroughly examines potential influences on vaccine hesitancy concerning individual/organizational, societal contextual, and vaccine/vaccination factors (14). Additionally, the 5C model of vaccine hesitancy links it to psychological factors and interprets its role from various dimensions (15). According to this model, factors such as the communication and media environment, regional culture, vaccine safety and efficacy, and trust in government and healthcare organizations are considered important in influencing vaccine hesitancy (16).

The transmission of vaccine-related information, including its source, demand, and operation, typically undergoes several stages before reaching the end-user. It’s crucial to recognize the information asymmetry inherent in this process, where both information providers and recipients may not have equal access to or possession of all necessary information for informed decision-making (17). This information gap, compounded by potential challenges related to digital literacy and internet usage among certain middle-aged and older adult individuals, renders them susceptible to encountering information barriers or delays. Consequently, this impedes their ability to trust medical information (18).

To gain insights into the current landscape of herpes zoster vaccine hesitancy among individuals aged 50 and above in China, and to develop strategies for enhancing vaccination and immunization planning, this study will combine the vaccine hesitancy determinants matrix with the 5C model. Through qualitative exploration, we will investigate the factors influencing herpes zoster vaccine hesitancy from the perspectives of individuals aged 50 and above, as well as healthcare professionals. The goal is to understand the influence of information asymmetry in this context and devise strategies to mitigate it.

## 2 Materials and methods

### 2.1 Study design

**Figure 1:**
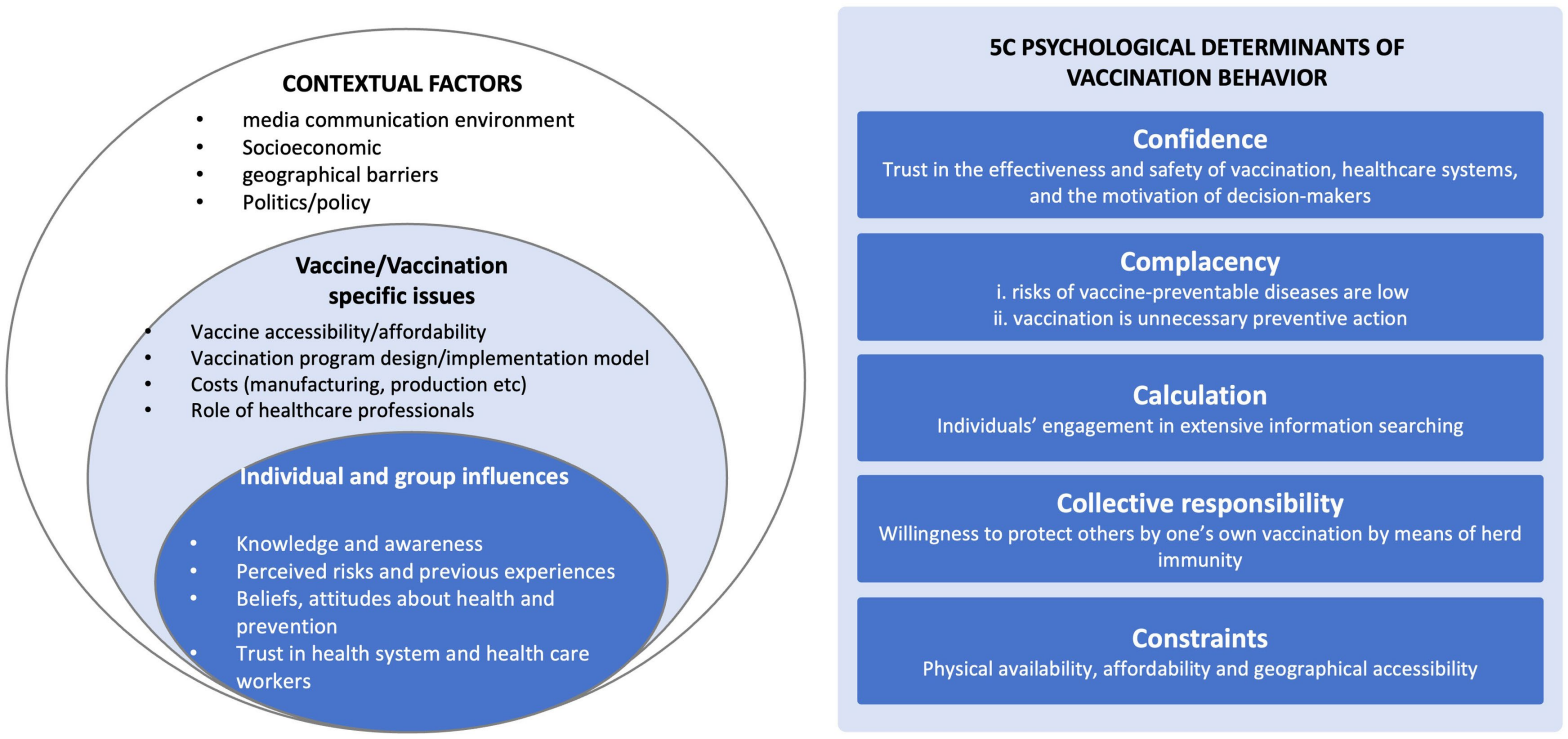
Vaccine hesitancy determinants matrix and the 5C model.

Following the methodology of descriptive research, we gathered data through face-to-face semi-structured in-depth interviews. Initially, we developed an interview outline based on the WHO Vaccine Hesitancy Determinants Matrix and the 5C model (Figure 1), aligning with the study’s objectives and literature review. The research design, analyses, and findings were reported following the Consolidated Criteria for Reporting Qualitative Research (COREQ) (19).

### 2.2 Participates

From October 2023 and December 2023, we employed purposive sampling at two community health centers in downtown and suburban Shanghai, China. Eligible participants were required to meet the following criteria: (1) aged ≥50 years, (2) residing in the community for ≥6 months, (3) capable of clear communication, and (4) providing informed consent. The study excluded people with poor health status unfit to participate in, as well as those with cognitive impairments that prevented from providing accurate and clear information. To comprehensively capture factors influencing vaccine hesitancy, we did not distinguish between participants based on herpes zoster vaccination status. Additionally, medical staff from both communities were recruited to participate in the survey, contributing to the comprehensive analysis. We conducted 17 in-depth interviews. The sample size was determined based on achieving thematic saturation, where no new themes were emerging from the data. Saturation was assessed through iterative data collection and analysis until consistent themes were identified. Specifically, the team conducted discussions to assess whether new interviews continued to contribute to understanding key themes related to vaccine hesitancy. We also employed thematic coding to monitor the emergence and repetition of themes. The decision to conclude data collection was based on the absence of new major themes (Supplementary material).

### 2.3 Data collection

Adhering to the principle of informed consent, prior to conducting the interviews, participants were provided with a detailed explanation regarding the purpose, methodology, and content of the interviews. The researcher had prior involvement in research projects on qualitative research at Fudan University and the University of Cambridge (online). Interviews were conducted in Chinese Mandarin in a comfortable, private setting, lasting 20 to 30 min each. All interviews were audio-recorded with participant consent. The research adhered to informed consent principles, with assurances of confidentiality and anonymity. Ethical approval was obtained from the Public Health and Nursing Research Ethics Committee of Shanghai Jiao Tong University School of Medicine (SJUPN-202018).

### 2.4 Data analysis

Transcriptions were completed within 48 h of interviews by researchers XL and YF. NVivo 14.0 was used for data management. During the transcription process, ambiguous sections were referred back to the interviewees for clarification. Data analysis involved open coding to identify and label themes, which were then categorized into a framework based on the 5C model and SAGE matrix. Categories included contextual factors, individual and group-related factors, and vaccine-related factors. Disputes in data categorization were resolved with input from a third researcher, FQ.

## 3 Results

### 3.1 General information on interviewees

A total of 17 adults aged 50 years and older (N1 ~ N17) and 4 healthcare workers (M1 ~ M4) working in the community were recruited for the study. The sample of middle-aged and older adults came from both urban and rural areas, comprising 5 males (29.4%) and 12 females (70.6%). The four healthcare workers’ job scope covered administration, general practitioner, and public health nursing. (See Supplementary Appendix 2 for more details about the participants).

### 3.2 Themes

Within the context of the three broad themes of the VHDM-individual and group influences, contextual factors and vaccine/vaccination-specific issues-the analysis yielded 10 themes (Table 1).

**Table 1:** Overview of themes.

Categories	Theme	Subtheme
Contextual factors	Access to the Internet and social media	Negative information
		Credibility of information
		Official authoritative information
	Community vaccination service	Proactive awareness
		Inconvenience of vaccination
	Socio-economic circumstances	Disparities in vaccine allocation
		Social insurance coverage
Individual and group influences	Disease and vaccine perception differences	Unfamiliar medical terminology
		Less information access initiative
	Negative disease experience and vaccination experience	Negative vaccine incidents
		Positive vaccination experience
	Vaccine complacency and risk perception	High vaccine complacency
		Misalignment of risk and benefit assessment
	Natural immunity preference	Preventing external vaccine damage
		Poor physical fitness
Vaccine/vaccination specific issues	Vaccine supplier experience	Vaccine supply source effects
		Attention from medical personnel
	Vaccination costs	Pressure on vaccine prices
		Health service prioritization
	Vaccination program design/implementation model	Optimization of vaccination plan
		Inadequate follow-up medical care

#### 3.2.1 Contextual factors

##### 3.2.1.1 Access to the internet and social media

The Internet and social media serve as crucial channels for information dissemination. However, they can also be platforms for spreading misinformation, exaggerations, or negative narratives, thereby exacerbating vaccine apprehensions, and fostering vaccine hesitancy.

*According to phone information, this vaccination is more likely to be contaminated, and I’m not sure if the imported vaccine is suitable for Chinese people… this condition appears to be merely suffering. (N17).*

Furthermore, the information offered via official channels may be insufficient to meet the needs of the public, or official information may not be disseminated as quickly or in the same manner as social media. This may result in a higher reliance on social media or other unofficial routes for information.

*I do not know that the community has publicized this, but neighborhood councils are allowed to take on the job of publicizing vaccines… such as the effectiveness of the vaccine, the price, and where to get vaccinated. (N3).*

*I did not notice a news program pushing people to obtain this vaccine… Older people like me who aren’t as familiar with cellphones tend to glance at roadside advertisements more. (N7).*

There are fewer sources of information in the official media, making it difficult for citizens to determine which information is real and reputable, and some may not be authoritative enough.

*Some official media, such as the Health Commission and the CDC, can use tiktok or WeChat platforms to advocate vaccination among the public. (N16).*

##### 3.2.1.2 Community vaccination service

The absence of supply-side information in the community, which is the primary source of firsthand and reliable information, may result in poor public understanding of the herpes zoster vaccine and its immunization services.

*I have not been informed about the availability of HZV in my neighborhood. Before the COVID-19, the community used to call me whenever there was a need for vaccination. (N12& N17).*

Inadequate or unavailable community-based vaccination services can hinder herpes zoster vaccination. Factors such as long distances to vaccination sites, inflexible vaccination scheduling, or insufficient vaccination staff create significant constraints. These constraints, as described in the 5C model, can all impact an individual’s decision regarding vaccination.

*This is the closest community where I can obtain HZV… I still hope that the country would increase the vaccine’s popularity and provide adequate support to people who wish to get vaccinated. (N3).*

Individuals with limited access to community vaccination information may turn to unreliable sources such as word of mouth, unofficial channels, or unconfirmed information. This lack of reliable information undermines their confidence in the vaccination process, leading to uncertainties and concerns.

*If the community had not pushed and publicized it with the necessary text messages, I would not have known about it. (N5).*

*I came to my community to inquire about the shingles vaccine after reading about it online, and I’m not sure whether what the internet claims is accurate. (N16).*

##### 3.2.1.3 Socio-economic circumstances

Individual immunization may be more convenient in urban regions due to the higher accessibility of healthcare resources and services. In contrast, suburban or economically disadvantaged communities may lack proper health care or immunization locations.

*Our community is one of the only locations in the surrounding community centers to offer the hzv, and we will increase the number of vaccines in time to meet the public’s demand. (M1).*

*Compared to other immunization program vaccines, this community hospital has minimal interactions regarding shingles visits… If you require the herpes zoster vaccine, schedule an appointment at a tertiary care facility. (M4).*

HZV is currently not covered by Medicare, which makes some seniors more hesitant to acquire it because they will have to pay for it themselves.

*This vaccination is quite expensive and does not qualify for Medicare. The older adult are more likely to get this illness, and many of them are retired and have limited resources. (N11).*

*I would absolutely consider getting the vaccination if it was reimbursable, like the COVID-19 vaccine, which is free, or reasonably priced, like the flu shot. (N15).*

#### 3.2.2 Individual and group influences

##### 3.2.2.1 Disease and vaccine perception differences

Most middle-aged and older persons have insufficient knowledge about vaccines and the diseases they protect against. In terms of disease awareness, respondents may be familiar with the symptoms of herpes zoster infection, such as “blisters” and “pain,” but they are unfamiliar with the disease’s precise name, how it is caught, and how it is treated.

*Is herpes like a snake wrapped around abdomen? It causes a red rash in a circle around the stomach and is extremely painful. (Key information-N13, N15, N17).*

Most urban inhabitants have only heard of vaccines by name and rarely initiate conversations concerning vaccines and diseases. Lack of initiative in information acquisition is a significant predictor of vaccine reluctance.

*I had no idea that this illness had a vaccine? I’m more inclined to go when the community suggests that a vaccine is accessible than when I proactively inquire. (N4).*

*I visit the neighborhood hospital primarily for medical examinations, immunizations are not very important to me. (N12).*

##### 3.2.2.2 Negative disease experience and vaccination experience

Patients who have been infected with shingles, or who have had the disease in close relatives or friends, are more aware of the disease’s seriousness and impact, and are thus more willing to be vaccinated to prevent recurrence, while also being more concerned about the vaccine’s efficacy and reliability.

*My parents and classmates have contracted this sickness, and the symptoms include severe discomfort… I’m willing to be vaccinated against this virus because it is vaccine-preventable, if the vaccine is effective. (N3).*

Individuals who have had an active vaccination experience may be more likely to acquire HZV and have greater confidence in its safety and efficacy.

*I’ve had the flu shot a few times, and I believe the vaccine is tested before it’s made available to the public, so I’m willing to receive the hzv as well. (N5).*

Individuals who have suffered adverse effects from previous immunizations may carry this worry over to the shingles vaccine, where they are more concerned about potential side effects.

*I experienced a fever after the COVID-19 vaccination previously, and because side effects might occur with a vaccine like this one that everyone is getting, I’m not sure how HZV will affect my health. (N1).*

##### 3.2.2.3 Vaccine complacency and risk perception

Higher vaccination complacency in middle-aged and older populations refers to a person’s confidence in their pre-existing immune system, believing that their body is already capable of fighting off diseases and possible infections. This sense of complacency, which is a key component of the 5C model, leads individuals to believe that shingles do not constitute a severe threat to their health. As a result, individuals with higher levels of complacency are hesitant to be vaccinated.

*The reason I did not think about it is that I have a very low probability of getting sick, and I have not had an infection at my age. (N9& N10).*

People tend to believe that treatment is more expensive than vaccination, underestimating their chance of contracting the illness and having insufficient knowledge about the advantages and effectiveness of HZV, both in terms of its protective effect and financial benefits.

*I used ointment to the rash every day after the infection, and it did not take much money… I do not think the vaccine is necessary. (N2& N9).*

##### 3.2.2.4 Natural immunity preference

Those who support natural immunity may be more likely to employ natural methods to increase the immune system, believing that naturally acquired immunity is more long-lasting and effective, and that external immunizations are hazardous.

*A vaccine is always something else shot into the body, and something foreign is damaging to the body. (N12).*

*Herpes zoster is thought to appear when you have a weakened immune system, thus I believe it is best to rely on your immune system. (N13).*

In addition to preferring autoimmunity against viruses because they are concerned about the harm caused by external immunizations, persons with objectively lower body mass prefer natural immunity to avoid difficulties and consequently lower health.

*I’ve had chronic nephritis and proteinuria for a long time… Personal health is a major reason why poorer health is not appropriate for immunizations. (N8).*

#### 3.2.3 Vaccine/vaccination specific issues

##### 3.2.3.1 Vaccine supplier experience

Healthcare providers-pharmaceutical corporations, government agencies, and public health organizations, play a critical role in vaccine supply and management, as well as vaccination service delivery. The imported recombinant herpes zoster vaccine is now approved in China, and while the indigenous vaccine is available in 2023, the two vaccines differ significantly in terms of efficacy, target demographics, and vaccination protocols. According to studies, the source of vaccine supply influences an individual’s likelihood of receiving the vaccine, with more participants showing greater trust in imported vaccines.

*Domestic vaccine appears to have been out only last year, whereas the imported vaccination is already accessible in so many nations. I would favor the foreign vaccine. (N3).*

*Currently, only imported shingles vaccination is accessible in our area. Although the domestic vaccine increases the age range for vaccination to over 40, the current guideline recommendation remains the imported recombinant shingles vaccine. (M1).*

Although compared to the middle-aged and older adult populations, the healthcare professionals interviewed did not exhibit significant vaccine hesitancy toward the herpes zoster vaccine, it is noteworthy that only one individual had actually received the vaccine. Even among medical professionals, awareness and prioritization of the herpes zoster vaccine remain insufficient. This lack of attention and expertise regarding the vaccine raises concerns about the accuracy of the information provided, resulting in insufficient access to vaccination information for the public and influencing their decision-making.

*My wife had gone to the hospital for herpes a few times before, but the physicians did not know much about the disease and did not recognize it as shingles at first. (N9).*

*I’ve got two vaccinations, but I’m not completely aware of the vaccine information… Indeed, as the group of persons in the community who have the most contact with older people, all community health professionals should be gathering to learn and train. (M2).*

*My expertise of shingles may be restricted to symptoms, location, and so on…*. *When someone asks about the content, there is no way to provide accurate information or fast immunization assistance. (M3).*

##### 3.2.3.2 Vaccination costs

The immunization cost of approximately 3,200 yuan for two doses may increase the financial burden on the middle-aged and older adult. This could be a major reason for vaccine hesitancy, particularly among individuals with low incomes or who are retired.

*My prior immunization cost roughly 3,000 RMB, which is quite pricey, and this vaccine is not covered by health insurance, unlike the flu vaccine, which I can probably obtain for a couple of hundred dollars. (N5).*

For interviewees with high incomes, the vaccine’s cost was not a big impediment. Rather, the vaccine’s effectiveness and hazards were important issues.

*Price does not matter to me if the outcomes are good enough and the side effects aren’t too awful, I’m willing to get immunized. (N4).*

Respondents from the suburbs stated that high-cost immunizations constitute an additional burden for families, which is likely to be a substantial role in vaccine hesitation. Still, if the shingles vaccine was free, like the COVID-19 vaccine is, more people would consider taking it.

*This price is always slightly more for the older adult, because we are more remote, and no one makes much money… If the state implements a policy allowing the older adult to get for free, there will undoubtedly be many eager participants. (N16).*

Individual health service priorities vary, and some middle-aged and older persons may allocate their limited health-care resources to more urgent or severe health issues rather than immunizations.

*There is a significant difference between 200 and 3,000 RMB, and with two shots costing me roughly half a month’s pension, I’d much rather spend the money on useful treatments or vaccinations for diseases with a larger incidence, such as the flu. (N1).*

##### 3.2.3.3 Vaccination program design/implementation model

The absence of large-scale immunization campaigns or poorly designed/implemented models may lower middle-aged and older persons’ desire to accept the shingles vaccine. For example, immunization sites may be remotely located, making transportation problematic; or vaccination schedules may be less frequent and prevalent than for the COVID-19 vaccine or flu vaccine.

*HZV is not as well-known as the flu or the COVID-19 vaccine, and we will often warn older adult individuals attending our clinics that ‘flu season’, ‘pneumonia is common’,* etc.*, but there is actually very little discussion of shingles…*. *The community currently offers vaccine appointments, but there are no large-scale programs to promote and vaccinate. (M2).*

*There are relatively few shingles vaccination campaigns or programs in the suburbs, and as a healthcare practitioner, I am aware of only a few community hospitals in urban regions that offer vaccinations. As a result, in addition to internal community awareness initiatives, all parties should work together to publicize the campaigns at the social and governmental levels. (M4).*

The adequacy of follow-up medical protection also influences the public’s motivation to get vaccinated. If people experience unpleasant responses or require more medical care following vaccination, a lack of proper support and protection may diminish their willingness to get vaccination.

*Post-vaccination complications are a concern…*. *I think the community must do a good job with post-vaccination because a lot of people get vaccinated for the first time, and if anything, horrible happens instead, it’s a method of undermining people’s trust. (N10).*

*Not only HZV, but also the community is now actively improving the process of immunization planning vaccination, from vaccine appointment to post-vaccination observation, and I hope that adequate support can be given, which is also a guarantee for the public’s safety. (M1).*

## 4 Discussion

**Figure 2:**
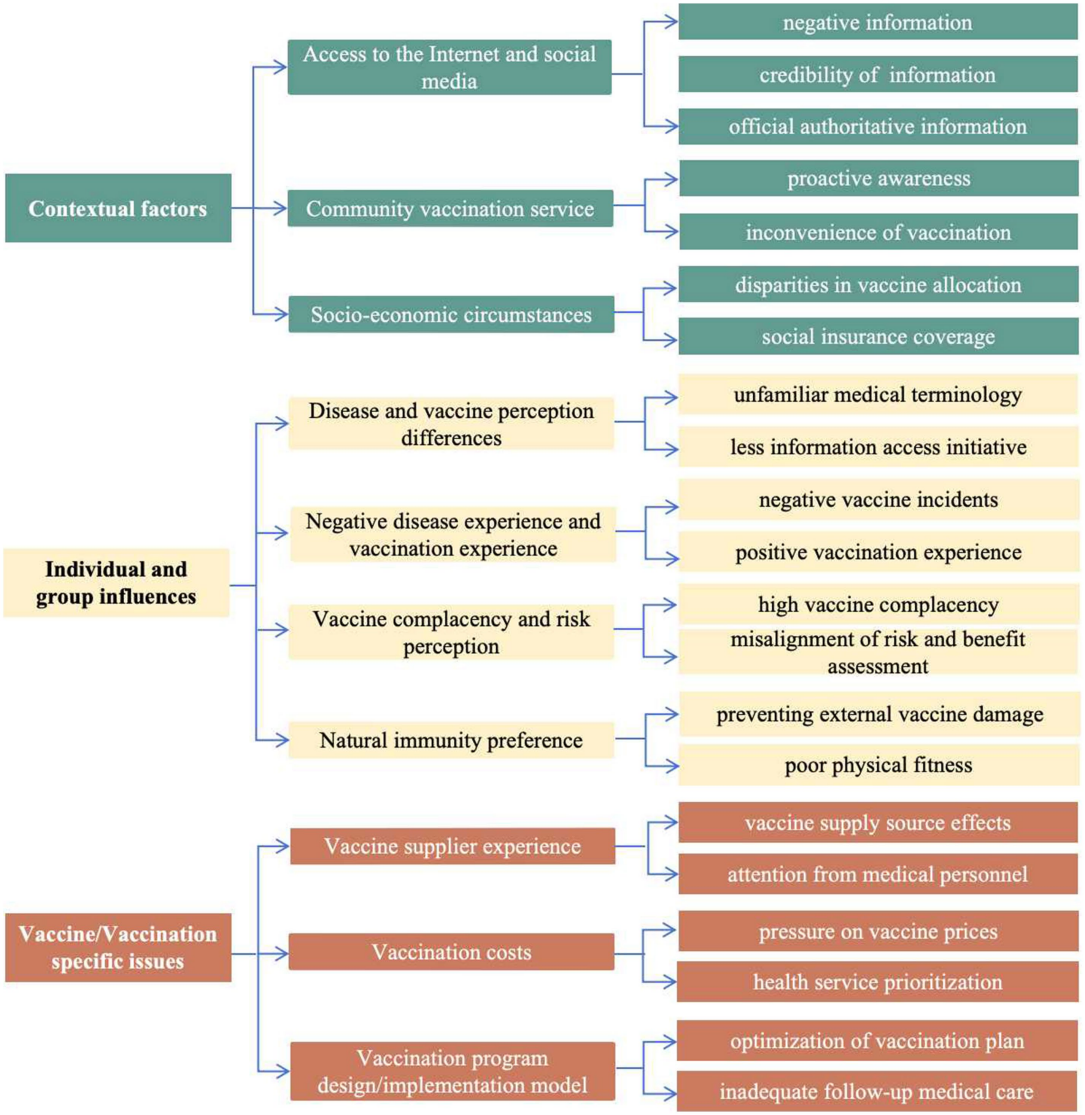
Visualization of interview themes.

**Figure 3:**
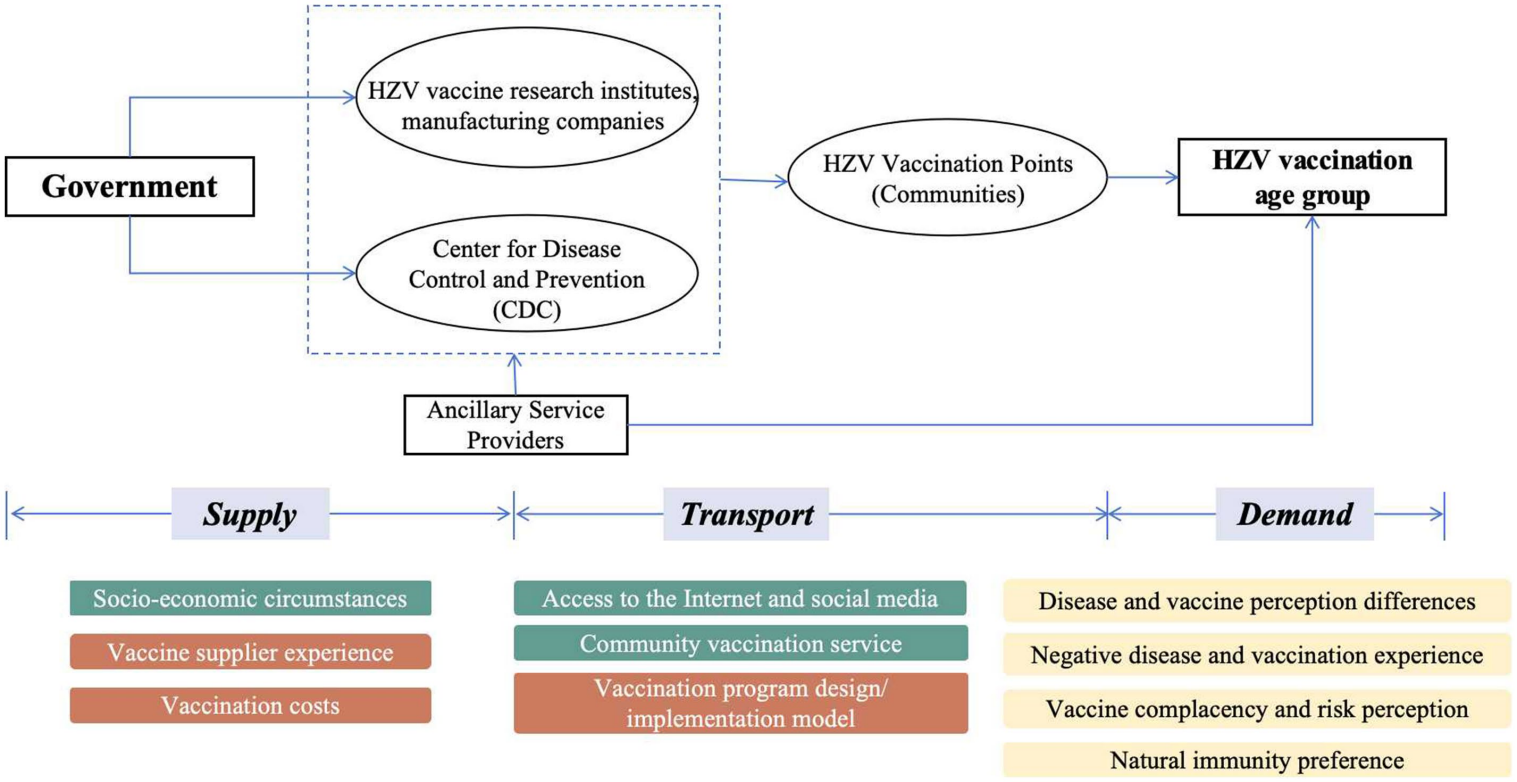
Vaccination processes and factors.

The 5C model and the vaccine hesitancy determinants matrix have been used in research on influenza (20), COVID-19 (21), and Monkeypox (22) vaccines to explore and organize influencing factors. In this study, these frameworks were applied to examine information asymmetry in the formation of herpes zoster vaccine hesitancy among adults aged 50 years and older across different vaccine processes (Figures 2, 3). Individual/group-level impacts include disparities in disease and vaccine perceptions, bad disease and vaccination experiences, vaccine complacency, and natural immunization preferences. Access to the Internet and social media are among the most important contextual factors, as they can contribute significantly to negative information provision and a lack of official authoritative information, as well as inconvenience and instability in community-based vaccination services and socioeconomic differences. Vaccine considerations encompass factors such as the experience of vaccine suppliers, costs, and the inadequate dissemination of essential vaccination information. Inequities and differences in the availability of information at various levels—ranging from broad external sources to individual access—significantly contribute to vaccine hesitancy.

### 4.1 Personalized vaccine information support to improve cognitive level and information acquisition initiatives for the middle-aged and older adult population

Several sub-themes, including cognitive variances, past vaccination experiences, and preferences for natural immunity, are encompassed within the factors influencing individuals and groups. Prior studies underscore the pivotal role of trust and mistrust in vaccine hesitancy when making vaccination decisions (23). Trust serves as a linchpin for individuals and communities to heed regulatory agency and medical personnel recommendations. However, establishing trust necessitates information acquisition, where individuals must decide whether to place trust or distrust based on the information received (24). Therefore, rectifying information asymmetry and addressing information gaps are pivotal in tackling herpes zoster vaccine hesitancy among the middle-aged and older adult population in China. Our findings indicate that individuals harboring lower levels of trust in authorities and healthcare providers may exhibit reluctance toward embracing new or relatively unfamiliar vaccines due to previous negative disease encounters or vaccine-related incidents, aligning with prior research (25). Those personally afflicted by herpes zoster, exposed to accounts of its agonizing symptoms, or having vaccinated family members or acquaintances are more inclined to comprehend the vaccine’s significance and benefits, thereby manifesting more favorable vaccination attitudes (26). Furthermore, individuals adopting a complacent stance toward the herpes zoster vaccine often showcase heightened levels of vaccine hesitancy, consistent with Abdou et al.’s (27) findings. Complacent individuals may perceive vaccination as superfluous, relying instead on their immune system’s defense mechanisms and shunning foreign vaccine interventions (28). Even after elucidating the vaccine’s active constituents, a significant portion of individuals still misconstrues vaccination as akin to inoculating the virus itself, potentially triggering the disease.

Understanding the expected benefits of vaccination through accurate information is essential in shaping vaccine attitudes. When individuals receive reliable information about how vaccination can effectively reduce the risk of infection, they are likely to have lower vaccine hesitancy (21). In numerous studies, ‘misinformation’ (29), ‘information gap’, and ‘trust deficit’ (18) stemming from negative information have been pinpointed as primary causes of hesitancy regarding various vaccines, such as the influenza vaccine and the COVID-19 vaccine. However, the shingles vaccine remains unpopular among Chinese adults aged 50 years and older (8). The emergence of information asymmetry due to information scarcity is gradually becoming a salient characteristic of the healthcare service landscape. The specialized nature of medical services coupled with the high cost of acquiring information among the middle-aged and older adult population has rendered many respondents’ passive recipients of medical services. Insufficient information significantly influences individuals’ trust levels and propensity to vaccinate (30). Therefore, enhancing publicity and educational efforts for herpes zoster vaccination is crucial for preventing and reducing vaccine hesitancy among middle-aged and older adult populations. Delivering personalized, easily comprehensible, scientifically accurate information in a manner that accommodates the information needs and preferences of the population could elevate disease and vaccine awareness levels. Consequently, improving access to accurate vaccine information may help correct misunderstandings and encourage more people to get vaccinated.

### 4.2 Access to the internet and social media diversifies authoritative information and increases the reliability of sources

Addressing the unique causes of vaccine hesitancy needs not only an individual focus, but also the collaboration of communities, health institutions, social media, and outside influences. The rise of social media has revolutionized information distribution, enabling both accurate and misleading information to spread rapidly (31). While social media offers a new and expansive platform for information dissemination, it has also allowed anti-vaccine advocates to reach and influence a large audience more effectively than traditional channels (32, 33).

The study showed that a lack of formal authoritative information can make it difficult for people to access correct and reliable vaccine-related information. When people want to learn about the vaccine’s efficacy, side effects, or the target population, they may rely on WeChat text messages, television advertisements, and other news and social media if they cannot get accurate information from official authoritative sources, which may be misleading or inaccurate, adding to people’s doubts and confusion about vaccination (18). As indicated by a study from India (21), respondents who trusted information shared by family members, friends, relatives, and health workers exhibited less vaccine hesitancy. In contrast, information from mass media and social media was associated with higher vaccine hesitancy. Respondents in the survey also stated that a lack of ambiguity in official information may call into question its credibility and authority, contributing to distrust of vaccination. Second, due to information occlusion, access to community vaccination is limited, and the combination of insufficient vaccine supply and unstable information sources contributes to individual obstacles to immunization. As a result, government officials can provide accurate, easy-to-understand vaccine-related information by developing a clear information dissemination plan, using electronic platforms such as WeChat, official Twitter and TikTok accounts, organizing regional health lectures, and establishing special consultation hotlines and vaccine clinics in the community to answer vaccine questions (34, 35). The strategies described above may improve the accuracy and transparency of official authoritative information, reduce the information gap between officials and the public, and boost individuals’ willingness to be vaccinated.

Limited understanding of the herpes zoster vaccine among middle-aged and older adults further hinders immunization efforts. Many individuals are unaware of the vaccine’s benefits and its relationship to the disease. A systematic review (36) found that 67.1% of respondents from 17 countries had limited knowledge about the herpes zoster vaccine, including its onset, progression, and treatment options. Information occlusion and limited community vaccination access also pose obstacles to immunization. To address these challenges, government officials should implement strategies to enhance the accuracy and transparency of vaccine information. This includes developing a clear information dissemination plan, utilizing electronic platforms such as WeChat, official Twitter and TikTok accounts, organizing regional health lectures, and establishing consultation hotlines and vaccine clinics. These measures can help bridge the information gap, improve public trust, and increase vaccine uptake.

### 4.3 Increasing the emphasis of community vaccine suppliers and accelerating vaccinations at the last mile

Interviews with community healthcare workers revealed that, compared to the middle-aged and older adult populations, they were more supportive of herpes zoster vaccination. This may be due to their frequent exposure to vaccines in their professional roles (37). However, the actual vaccination rate among healthcare workers was low. This discrepancy can be attributed to two main factors. Firstly, unlike the COVID-19 vaccine, there are no mandatory regulations for herpes zoster vaccination in China (38), which means there are no clear requirements or guidelines for its administration. Secondly, a lack of awareness and understanding about the herpes zoster vaccine persists, even among medical professionals (39). Our study found that healthcare workers had gaps in their knowledge about herpes zoster, and they had received minimal specialized training or education on the subject, with few professional courses on herpes zoster available.

As a result, healthcare providers are unable to effectively communicate the importance of the herpes zoster vaccine, leaving the public unaware or skeptical about its benefits (40). Interviews with four healthcare professionals highlighted substantial gaps in their knowledge about herpes zoster in both suburban and urban communities. This lack of specialized courses and training results in many residents not receiving professional vaccine advice.

Previous research has indicated that healthcare professionals’ knowledge and guidance are critical for individual immunization (41). Because the community is the most convenient location for residents to seek medical care, it serves as the primary channel for disseminating authoritative information about vaccines. This setting is ideal for explaining the importance, safety, and benefits of vaccines, sharing actual vaccination experiences, and understanding patients’ specific needs and concerns (42, 43). However, due to China’s large older adult population and the high volume of community outpatient clinics, our study found that community physicians struggle to find time to address the vaccine needs of the middle-aged and older adult populations. Therefore, it is recommended to establish specialized clinics with dedicated staff to efficiently recommend various types of vaccines (44).

In addition, vaccine affordability, including the cost and individual economic circumstances, may contribute to vaccine hesitancy among the middle-aged and older adult. In this survey, a number of respondents indicated that the high price may be an important reason for discouragement after learning the relevant information, which is consistent with the findings of a Hong Kong study (26), in which knowledge of the disease and the vaccine played a secondary role in vaccination decision-making when compared to the economic burden of the herpes zoster vaccine. In China, the herpes zoster vaccine is currently available only through self-funded, voluntary vaccination. There are no conditions for providing the vaccine free of charge at this time. However, offering economic incentives might be a feasible alternative. For instance, community-level support could include providing daily necessities or vouchers as incentives for vaccination (45, 46).

This study had limitations. First, the participants were middle-aged and older individuals from two community health centers in both urban and suburban areas of Shanghai. The relatively small sample size and localized focus may restrict the generalizability of the findings and not capture all factors related to vaccine hesitancy. Second, while the study highlights the role of information asymmetry and examines the perspectives of middle-aged and older adults as well as healthcare providers, it does not include viewpoints from leaders of National Health Commission or relevant government departments. Finally, the study employs qualitative methods, focusing on in-depth interviews and thematic analysis. As such, the findings are illustrative rather than universally applicable. Future research should consider incorporating a broader range of data sources and methods to enhance the generalizability and applicability of the conclusions.

## 5 Conclusion

Herpes zoster vaccine hesitancy remains a significant issue among middle-aged and older adults in China. Most individuals lack understanding of vaccination-related information, compounded by problems of information asymmetry, vaccine complacency, and inadequate community services. Vaccine hesitancy is influenced by various factors such as online information, individual vaccination history, and trust levels. These findings suggest that public health departments can reduce individual cognitive bias, overcome information gaps and disparities, and promote higher vaccination rates by disseminating diverse and credible vaccine information through multiple channels, including social media, medical institutions, and offline contact.

## Data Availability

The raw data supporting the conclusions of this article will be made available by the authors, without undue reservation.
