# Supplementary material for: Understanding herpes zoster vaccine hesitancy and information asymmetry: a qualitative study in China
Source: Front Public Health. 2024 Sep 2;12:1429522. doi: 10.3389/fpubh.2024.1429522 (PMC11402811; doi:10.3389/fpubh.2024.1429522)
Supplement: Supplementary file 1 [file Data_Sheet_1.docx]

***Supplementary Material***

**1 In-Depth Interview Guide**

**1.1 General information questionnaire**

No._____ Gender: _____ Age: _____ Education Level: _____

**1.2 Interview Guide**

Date: ___month ___day ___year Community: _____

Welcome: *Good morning (afternoon), my name is xxx, and we are conducting a study on shingles vaccine hesitancy in community-dwelling middle-aged and older adults aged 50 years and older. By talking with you, we hope to understand the specific content and manifestation of the hesitancy of the herpes zoster vaccine in the middle-aged and elderly population, which involves the doubts and attitudes towards the herpes zoster vaccine, etc. The results of this study will help experts in the field, medical staff of community health centers, etc. to raise the level of attention to the situation of the herpes zoster vaccine in the community, to lay the foundation for providing more professional and detailed preventive vaccination services. With your consent, I will record our conversation to facilitate clearer documentation of all the details of the conversation. All data will be coded and displayed anonymously.*

**2 Supplementary Figures and Tables**

**Table S1. Outline of interviews with adults aged 50 and over and healthcare professionals.**

| ≥ 50 years old | | Community health workers |
| --- | --- | --- |
| 1 | Describe what you know about herpes zoster? | What do you think are the effects of shingles on middle-aged and older adults? |
| 2 | What do you know about the herpes zoster vaccine? | What do you know about *HZV*? |
| 3 | Have you ever considered getting *HZV^1^*?  *If so, what factors influenced your decision?* | What information about *HZV* have you shared with middle-aged and older adults? |
| 4 | What are your fears and concerns about getting *HZV*? | How do you think it would be more effective to promote vaccine information in your community? |
| 5 | Has anyone, organization or community ever given you information about *HZV*? | What do you think are the main factors influencing *HZ* vaccination in middle-aged and older adults? |
| 6 | Are you aware of the state and government's opinion on herpes zoster vaccination for middle-aged and older adults?  *Does their recommendation or lack thereof influence your intention to receive shingles vaccination? affect your intention to be vaccinated against HZ?* | What role do you think healthcare professionals play in promoting the herpes zoster vaccine? |
| 7 | What do you think the cost of HZV would be for you to consider getting it? | Have you discussed herpes zoster (vaccine) with others working in the medical field? |
| 8 | Have you spoken with a healthcare professional about *HZV*?  *Did their opinions influence your decision?* | What steps do you think can be taken to promote herpes zoster vaccination among 50-year-old and above? |
| 9 | What would you like to see communities or healthcare organizations do to promote vaccination rate of herpes zoster? |  |

*Note.* HZV: Herpes Zoster Vaccine

A total of 17 adults aged 50 years and older (N1~N17) and 4 healthcare workers (M1~M4) working in the community were recruited for the study. Specific results are shown in ***tables S2 & table S3*** below.

**Table S2.** Details of the study participants (above 50 or older, *n*=17)

| No. | Gender | Age | Education level | income per month (CNY) | chronic disease | Residency | HZ^c^ vaccination status | HZ Vaccine Hesitancy |
| --- | --- | --- | --- | --- | --- | --- | --- | --- |
| N1 | female | 74 | junior college | 5000～10000 | hypertension | urban^a^ | / | yes |
| N2 | female | 67 | high school | 5000～10000 | / | urban | / | yes |
| N3 | male | 55 | junior college | >10000 | / | urban | second shot | no |
| N4 | female | 63 | junior college | >10000 | / | urban | / | no |
| N5 | male | 75 | undergraduate | >10000 | diabetes | urban | second shot | no |
| N6 | female | 73 | undergraduate | >10000 | / | urban | second shot | no |
| N7 | female | 66 | junior high school | 3000～5000 | / | urban | / | yes |
| N8 | female | 69 | junior high school | 5000～10000 | chronic nephritis | urban | / | yes |
| N9 | male | 81 | high school | 3000～5000 | hypertension | urban | / | yes |
| N10 | male | 67 | junior high school | 3000～5000 | / | urban | / | yes |
| N11 | female | 72 | junior high school | 5000～10000 | / | urban | / | no |
| N12 | female | 78 | primary school | 1000～3000 | hypertension | suburb^b^ | / | yes |
| N13 | female | 62 | high school | 1000～3000 | / | suburb | / | yes |
| N14 | female | 65 | junior high school | 3000～5000 | / | suburb | / | no |
| N15 | female | 59 | junior high school | 5000～10000 | / | suburb | / | yes |
| N16 | female | 57 | junior high school | 3000～5000 | / | suburb | / | no |
| N17 | male | 58 | junior college | 5000～10000 | / | suburb | / | yes |

*Note*：a- Urban, Xuhui district, central Shanghai; b- suburban, Fengxian suburban district of Shanghai; c- HZ, herpes zoster.

**Table S3.** Details of the study participants (health care worker, *n*=4)

| No. | Gender | Age | Education level | Title | Profession | location | HZ vaccination status | HZ Vaccine Hesitancy |
| --- | --- | --- | --- | --- | --- | --- | --- | --- |
| M1 | female | 42 | master | advanced | administration | urban | / | no |
| M2 | female | 32 | master | medium | general physician | urban | second shot | no |
| M3 | female | 39 | bachelor | medium | Nursing management | suburb | / | yes |
| M4 | male | 27 | bachelor | junior | public health nurse | suburb | / | no |
